# Comparison of Methods Study between a Photonic Crystal Biosensor and Certified ELISA to Measure Biomarkers of Iron Deficiency in Chronic Kidney Disease Patients

**DOI:** 10.3390/s17102203

**Published:** 2017-09-25

**Authors:** Ross D. Peterson, Kenneth R. Wilund, Brian T. Cunningham, Juan E. Andrade

**Affiliations:** 1 Department of Food Science and Human Nutrition, University of Illinois at Urbana-Champaign, Urbana, IL 61801, USA; rdpeter2@illinois.edu; 2 Department of Kinesiology and Community Health, University of Illinois at Urbana-Champaign, Urbana, IL 61801, USA; kwilund@illinois.edu; 3 Division of Nutritional Sciences, University of Illinois at Urbana-Champaign, Urbana, IL 61801, USA; 4 Department of Electrical and Computer Engineering, University of Illinois at Urbana-Champaign, Urbana, IL 61801, USA; bcunning@illinois.edu; 5 Department of Bioengineering, University of Illinois at Urbana-Champaign, Urbana, IL 61801, USA

**Keywords:** photonic crystal biosensor, iron deficiency biomarkers, method validation, total allowable error, analytical quality specification

## Abstract

The total analytical error of a photonic crystal (PC) biosensor in the determination of ferritin and soluble transferrin receptor (sTfR) as biomarkers of iron deficiency anemia in chronic kidney disease (CKD) patients was evaluated against certified ELISAs. Antigens were extracted from sera of CKD patients using functionalized iron-oxide nanoparticles (fAb-IONs) followed by magnetic separation. Immuno-complexes were recognized by complementary detection Ab affixed to the PC biosensor surface, and their signals were followed using the BIND instrument. Quantification was conducted against actual protein standards. Total calculated error (TEcalc) was estimated based on systematic (SE) and random error (RE) and compared against total allowed error (TEa) based on established quality specifications. Both detection platforms showed adequate linearity, specificity, and sensitivity for biomarkers. Means, SD, and CV were similar between biomarkers for both detection platforms. Compared to ELISA, inherent imprecision was higher on the PC biosensor for ferritin, but not for sTfR. High SE or RE in the PC biosensor when measuring either biomarker resulted in TEcalc higher than the TEa. This did not influence the diagnostic ability of the PC biosensor to discriminate CKD patients with low iron stores. The performance of the PC biosensor is similar to certified ELISAs; however, optimization is required to reduce TEcalc.

## 1. Introduction

Anemia is suspected in all chronic kidney disease (CKD) patients who are undergoing hemodialysis [1]. A functional or absolute iron deficiency is estimated in 25%–38% of CKD patients with anemia [2]. Despite the administration of recombinant erythropoietin (EPO, a renal hormone that stimulates red blood cell production), less than one-third of hemodialysis patients with CKD achieve target hemoglobin levels of 11 to 12 g/dL [3]. As a result, CKD patients with iron deficiency anemia (IDA) suffer secondary consequences leading to an increased risk of cardiovascular disease [4], cognitive impairments [5], and fatigue and mortality rates [6]. To improve delivery efficacy, intravenous (IV) iron treatments began in the early 1990s because they decreased the cost of EPO treatments to increase red blood cell levels [7] and improved other quality of life measures [8]. 

Iron staining on bone marrow aspirate smears is the gold standard for determining iron status; however, the invasiveness of this procedure limits widespread application [9]. Therefore, measuring protein biomarkers (i.e., ferritin and soluble transferrin receptor) offers the most practical route to diagnosing IDA in hemodialysis patients. IDA in CKD patients is diagnosed based on a protein biomarker and a relationship of biomarkers: serum ferritin and transferrin saturation (TSAT), respectively [10]. Ferritin is a large spherical protein that stores iron atoms within a cage-like structure [11], while TSAT is a non-protein biomarker that reflects the iron available for erythropoiesis. However, these biomarkers do not always deliver dependable results when working with CKD patients undergoing hemodialysis due to its inflammatory nature [10]. The soluble transferrin receptor (sTfR) is a truncated protein circulating in blood that originates from the transferrin receptor protein found within membranes of erythroid tissues [12]. The advantage associated with measuring sTfR is its stability during episodes of acute and chronic inflammation [13]. In spite of this advantage, measuring sTfR in hemodialysis patients to assess IDA has not been confirmed [14,15]. 

The primary diagnostic platform employed to measure protein biomarkers of IDA is the enzyme-linked immunosorbent assay (ELISA) [16]. ELISAs have high sensitivity due to the horseradish peroxidase reaction with a substrate that develops color and amplifies the signal detectable by a spectrophotometer. Although ELISAs are mainstream diagnostic tools, they are expensive and require laboratory equipment and trained personnel to run experiments. As an alternative, optical biosensor technologies based on photonic crystals (PCs) provide a facile mechanism for detection by harnessing the properties of light to achieve accurate and precise IDA biomarker detection [17,18]. PCs are periodic subwavelength grating structures that reflect a narrow band of wavelengths when illuminated by a broadband light source [19] at the resonance conditions. Biomolecule binding to the biosensor surface increases the refractive index of the resonance mode and results in a shift in the peak wavelength value (PWV) to a higher value. The amount of wavelength shift (ΔPWV) is directly proportional to the mass density of the molecules bound to the biosensor surface. This technique has been applied to biomolecule detection ranging from cells to viruses and pharmaceuticals [20,21,22,23]. Our recent studies measuring biomarkers of IDA contribute to our long-term goal of developing and validating PC biosensors for point-of-care (POC) diagnostic applications in which the detection instrument is a smartphone [24,25]. PC-based sensing platforms for clinical biomarkers benefit from cost-effective fabrication and short assay time. Some clinical applications include determination of sugars in blood and urine [26], cancer biomarkers [27], and immune thrombocytopenia [28]. The development of diagnostic tools for nutrition-related biomarkers and their clinical validation lags behind. 

Method validation studies provide the foundation for assessing the performance and acceptability of new diagnostic tools. Analytical quality specifications of more than 300 biomarkers have been published [29]. Analytical quality specifications set baseline levels of the total allowable error (TEa) that clinical diagnostic instruments can exhibit when measuring a biomarker of interest. Analytical specifications are critical to provide accurate, precise, and reliable diagnosis for patients in order to prevent misdiagnosis of disease or deficiency. As POC biosensors become available for clinical diagnostic applications, method validation studies are required to estimate their total analytical error. Error analysis is used to determine whether the total error of the proposed biosensor is acceptable at clinically relevant or cutoff concentrations. The certification of diagnostic devices is dependent on the total calculated error (TEcalc), which is quantified by replication experiments and comparison of methods experiments [30]. When the TEcalc is less than TEa for a given biomarker, the method is considered acceptable [29]. 

In previous studies, the accurate and reliable detection of ferritin on the PC platform followed a similar format to the standard ELISA (i.e., antigens within a sandwich of antibodies) [17]. A variation of this method capitalizes on the magnetic properties of functionalized iron oxide nanoparticles (fAb-IONS) to extract metabolites (i.e., sTfR) of interest first, and then measure their quantity on the PC platform [18]. This variation helped reduce interference signals caused by non-specific binding of proteins in a serum matrix—a common problem shared by all diagnostic platforms. Thus, this variation of the PC biosensor protocol might be useful to evaluate sera from CKD patients known to contain high levels of interference molecules due to the inability of the kidneys to effectively excrete endogenous [31,32] and exogenous compounds [33]. 

The great majority of studies often evaluate the performance of biosensors by diluting or dispersing the biomarker of interest in simple biological matrices such as buffer or bovine serum albumin, and rarely by measuring the serum of actual patients. Therefore, in the present study, the performance of the PC biosensor for the determination of ferritin and sTfR in CKD patients was determined. In addition, the total analytical error of the PC biosensor in quantifying IDA biomarkers was determined using FDA-certified ELISAs as the reference methods. 

## 2. Materials and Methods

### 2.1. Reagents

3-Glycidoxy-propyl-trimethoxy-silane (GTPMS) and NaOH were purchased from Sigma-Aldrich (St. Louis, MO, USA); phosphate-buffered saline (PBS) and StartingBlock blocking buffer were from Pierce (Thermo-Fisher Scientific, Waltham, MA, USA). Double deionized water (DDW) was used in all experiments. 

### 2.2. ELISA Kits, Standards, and Antibodies (Ab) Used in the PC Biosensor Assay

sTfR and ferritin ELISA kits were purchased from R&D Systems (DTFR1; Minneapolis, MN, USA) and ALPCO^®^ (25-FERHU-E01; Salem, NH, USA), respectively. Deidentified sera from hemodialysis patients—all of whom had end-stage renal disease (CKD stage 5)—were used in this study. Both monoclonal mouse anti-human capture (ab38168) and detection (ab10249) antibodies for the PC sTfR assay were purchased from Abcam^®^ (Cambridge, MA, USA). For the PC ferritin assay, capture (F4015; monoclonal mouse anti-human liver ferritin) and detection (F4015-17; goat anti-human liver ferritin) antibodies were purchased from US Biological. The detection antibody was conjugated with iron oxide nanoparticle conjugation kit from Ocean NanoTech (San Diego, CA, USA). Lyophilized forms of ferritin (94/572) and sTfR (07/202) were obtained from the National Institute for Biological Standards and Control, UK. These are World Health Organization (WHO) reference standards, and were diluted as recommended in PBS. A ferritin reference standard from the WHO was used to develop the standard curves to measure ferritin in the hemodialysis sera. Standards of sTfR were purchased from BioVendor™ (Asheville, NC, USA) and were calibrated with WHO recombinant sTfR reference reagent [18]. A serial dilution of ferritin (i.e., from 1000 to 62.5 ng/mL) and sTfR standards (i.e., from 6 to 0.25 µg/mL) were measured in triplicates in each plate. All sera samples were plated in triplicate as well.

### 2.3. PC Biosensor and Readout System

Detailed information about the PC biosensor platform and its readout system can be found in [19,34]. PC microplates (96-well) were purchased from SRU Biosystems, Inc. (Woburn, MA, USA). The Biomolecular Interaction Detection system (BIND; SRU Biosystems, Inc.) was used to detect biomolecule interaction on the biosensor surface (i.e., antibody interactions with antigens). The detection instrument illuminates the PC with a broadband light source (λ range 400–700 nm) that provides collimated light at normal incidence via an optical fiber positioned below the biosensor microplate and thus capable of measuring peak wavelength value (PWV) of a 96-well microplate in ~10 s. For all experiments presented using the PC biosensor, the quantities are reported as PWV shifts (nm).

### 2.4. Preparation of PC Biosensor

#### 2.4.1. Epoxy-Silanization of PC Biosensor Surface

The PC biosensor surface was functionalized similarly to the previously described method [17]. Briefly, each well was incubated for 1 h at 23 °C in a solution of 0.1 M NaOH, before sonication (Isotemp202, Thermo-Fisher Scientific) for 15 min. After aspiration and blotting, wells received 30 µL of 2.5% GTPMS and 10 mM acetic acid in ethanol solution and were incubated for 1 h at 23 °C. Lastly, wells were aspirated and washed twice with ethanol and dried under nitrogen stream before rinsing with phosphate-buffered saline (PBS) for assay preparation.

#### 2.4.2. Capture Monoclonal Antibody Immobilization

For each assay, 30 µL of capture antibodies for sTfR (40 µg/mL) and ferritin (100 µg/mL) was dispensed into all epoxy-silanized wells. The PC microplate was sealed with tape (Pierce, Thermo Fisher Scientific), incubated at 23 °C for 5 h, and then washed three times with PBS. The PWV corresponding to capture antibody immobilized on sensor surface was measured relative to baseline, which was measured right before the addition of the capture Ab. 

#### 2.4.3. Blocking Step

Undiluted StartingBlock (30 µL) was dispensed into all wells. PC microplates were incubated for 45 min at 23 °C and then washed with PBS three times. The PWV shift was measured relative to baseline and left ready to detect the complexes of fAb-IONs and antigens.

#### 2.4.4. Preparation of Functionalized Iron Oxide Nanoparticles (fAb-IONs)

Detection antibodies were functionalized (fAb) to iron-oxide nanoparticles (30 nm) by the vendor (Ocean NanoTech, LLC) and described in a previous study without modifications [17,35]. Conjugation was verified by gel electrophoresis from Ocean NanoTech (data not shown). Conjugations are expressed as mg of Fe per mL.

### 2.5. sTfR and Ferritin Detection Using PC Biosensor

This study used de-identified serum samples from a different study with kidney disease patients. Only a three-digit reference number which was not associated with any patient records was given to investigators. Briefly, blood samples were obtained from hemodialysis patients from their existing vascular access, immediately prior to a regularly scheduled hemodialysis session. Serum was collected, aliquoted, and frozen at −80 °C until analysis. Diluted (1:5 *v*/*v* in PBS) serum samples (60 μL) were mixed with either anti-sTfR (60 μL) or anti-ferritin (60 μL) fAb-IONs (1:2 *v*/*v*) for a final serum dilution of 1:10 *v*/*v* in 1.5-mL microcentrifuge tubes. Samples were then incubated at 23 °C on a shaker (400 rpm) for 1 h. Then, the tubes were placed in a SuperMag Multitube Separator™ (Ocean NanoTech) for 1 h until complexed fAb-IONs-antigen formed pellets along the side of the microcentrifuge tube. Serum supernatant was aspirated without disturbing the pellet, and the latter was reconstituted with PBS buffer (120 μL). Finally, the reconstituted samples were assayed in triplicate (30 μL/well) onto the PC biosensor plate and read immediately using the BIND instrument. Although detection signals from binding happened immediately and showed significant differences from controls after 20 min, signal was followed for 3 h at 1 min intervals. Due to the unknown components of some patients’ sera, some fAb-IONs formed aggregates after dispersion, which could not be reconstituted with PBS into a stable colloidal solution. These samples were not included in the final analysis of sTfR or ferritin. 

### 2.6. Determination of Inaccuracy and Bias in the Quantification of sTfR and Ferritin on the PC Biosensor and Comparison against Reference ELISAs

#### 2.6.1. Different Plots

Inherent imprecision was calculated using Equation (1):

(1) $$\sigma^{2}\left(\delta \right){=\sigma }_{T}^{2}{+\sigma }_{R}^{2}$$

where σ^2^T is the variance of the test method (i.e., PC) and σ^2^R is the variance of the reference method (i.e., ELISA) for each biomarker, and σ^2^(δ) is the total inherent imprecision of the test and reference methods together [36]. Difference plots were constructed to determine if the PC biosensor measured each biomarker statistically differently from the ELISA method. In this test, the null hypothesis was that the measured differences for all samples are zero and the constant analytical SD are presumed to be equal to σT + σR. Therefore, when the two methods are identical, it is expected that 68% of differences are distributed around 0 ± 1σ(δ), and 95% of differences are distributed between 0 ± 2σ(δ), as illustrated in the different plots [36].

#### 2.6.2. Systematic and Random Error

Mean differences between methods (i.e., systematic error or bias) were evaluated by a paired *t*-test and the distribution of differences (i.e., random error or the SD of differences) by an F-test. For the paired *t*-test, three statistics are calculated: bias, the SD of differences, and the *t*-value. Bias was calculated using Equation (2):

(2) $$\mathrm{bias}=\bar{y}-\bar{x}$$

where $\bar{y}$ is the mean of the PC biosensor method and $\bar{x}$ is the mean of the ELISA method. The SD of the differences (SDdiff) is calculated with Equation (3):

(3) $${\mathrm{SD}}_{\mathrm{diff}}=\sqrt{\frac{\sum^{\text{​}}{\left[\left(\mathrm{yi}-\mathrm{xi}\right)-\mathrm{bias}\right]}^{2}}{N-1}}$$

Finally, the *t*-value is calculated using Equation (4):

(4) $$t\;=\;\frac{\mathrm{bias}}{\frac{{\mathrm{SD}}_{\mathrm{diff}}}{\sqrt{N}}}$$

In which the *t*-value expresses the systematic error (bias) in multiples of random error (i.e., SDdiff). Critical values of *t* were selected at *p* = 0.05 and N − 1 degrees of freedom for each biomarker using a *t*-table [37].

F-tests in method validation studies are used to evaluate differences in random error between detection methods. F-test statistic was calculated using Equation (5):

(5) $$F=\frac{{{(s}_{1})}^{2}}{{{(s}_{2})}^{2}}$$

where s_1_ is the variance of the more imprecise method and s_2_ is the variance of the less imprecise method. Variance was calculated using Equation (6):

(6) $$s=\frac{\sum_{i=1}^{n}{{(x}_{i}-\mu )}^{2}}{\left(n-1\right)}$$

If both methods have equal variances, then F-test = 1. Critical values of F were selected at *p* = 0.05 and N − 1 degrees of freedom for the denominator and numerator for each biomarker using an F-table.

The total analytical error of the PC biosensor was calculated based on the systematic and random error using Equation (7):

(7) $${\mathrm{TE}}_{\mathrm{calc}}=\mathrm{SE}+\mathrm{RE}$$

where TEcalc is the total calculated analytical error of PC biosensor, SE is the systematic error, and RE is the random error. The TEcalc also included a factor of two to inflate random error as shown in Equation (8):

(8) $${\mathrm{TE}}_{\mathrm{calc}}{=\mathrm{bias}}_{\mathrm{meas}}{+2s}_{\mathrm{meas}}$$

This inflation of error in method validation is used to establish performance within acceptable limits of error [37]. Because the TEcalc was greater than the TEa, no other multipliers (e.g., 4s_meas_ or 6s_meas_) to inflate the error were tested [38]. The TEcalc was then compared to an established and agreed upon total allowable error. TEa is estimated based on within-individual and between-individual biological variation and clinical significance [39,40]. The TEa can be expressed as either an absolute concentration, as a percentage of a clinically relevant cutoff, as data average, or as a range determined by a survey group [37]. For sTfR, the TEa has been estimated as a percentage of a clinically relevant concentration, and for ferritin as data average.

## 3. Results and Discussion

### 3.1. Measured Means, SD, Coefficient of Variation, and Range of Sample Concentrations

**Figure 1:**
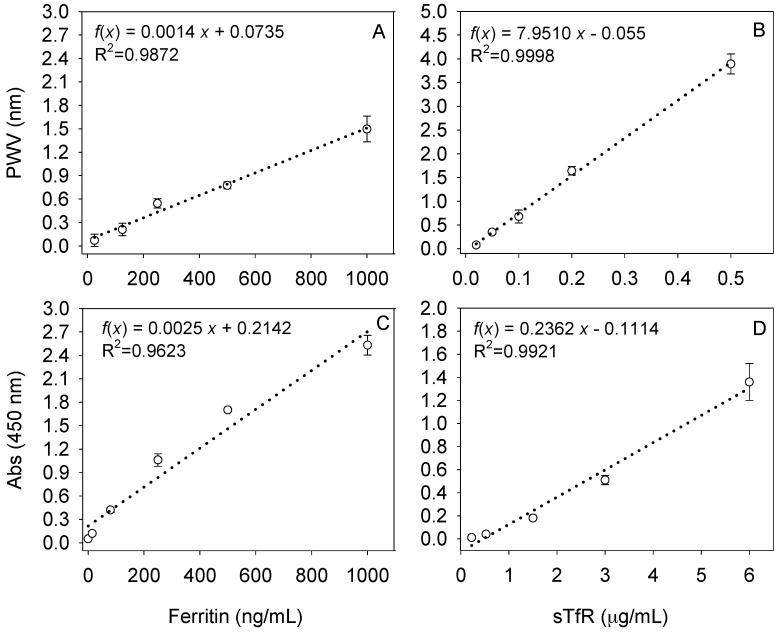
Standard curves for ferritin (left) or soluble transferrin receptor (sTfR; right) on (**A**,**B**) the photonic crystal (PC) biosensor or (**C**,**D**) certified enzyme-linked immunosorbent assays (ELISAs). PWV: peak wavelength value.

Both PC biosensor and ELISA platforms showed adequate linearity, specificity and sensitivity for standards as reported in previous studies and by the vendors (Figure 1). Results from precision studies and limits of detection (LODs) and quantification were reported previously. The dynamic range was assessed by testing several concentrations of the biomarkers of interest as suggested by others. The linear range was extracted from plotting and creating a linear equation with an R^2^ higher than 0.98 [41]. Limits of detection for ferritin (26 ng/mL) and sTfR (21 ng/mL) on the PC biosensor were calculated by estimating the noise signal arising from the blank and adding the SD of the lowest measured concentration of the biomarker of interest multiplied by 1.64 [42], and were similar as reported before [17,18]. 

Table 1 shows the mean, standard deviation (SD), coefficient of variation (CV), range of measurements, LOD, and dynamic range when quantifying serum sTfR and ferritin from CKD patients on each analytical platform. Biomarker means were similar (*p* > 0.05) when comparing the PC biosensor and the ELISA (sTfR: 1.21 vs. 1.30 µg/mL; ferritin: 279.5 vs. 276.8 ng/mL, respectively). The concentration ranges of sTfR and ferritin found in this study were similar to those reported by others for patients undergoing hemodialysis [15,43,44,45]. Furthermore, the SD and CV were not substantially different, although the ELISAs had less imprecision than the PC biosensor. However, these descriptive statistics do not provide sufficient information to qualify the PC biosensor as a reliable diagnostic platform. In method validation studies, it is more important to look at the individual sample differences between methods instead of the overall distribution around the mean. The wide range of measurements obtained in the ELISAs as compared to the PC biosensor might be indicative of other differences in measurement not apparent in descriptive statistics. 

### 3.2. Difference Plots Comparing Mean Differences When Measuring Ferritin and sTfR on the PC Biosensor and Reference ELISAs

**Figure 2:**
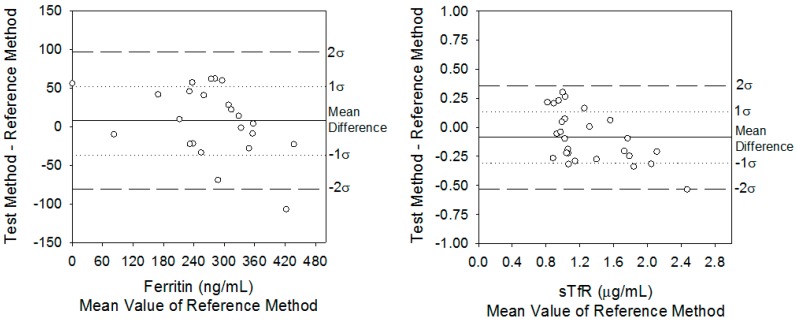
Difference plots comparing serum ferritin and sTfR concentrations from hemodialysis patients using the PC biosensor against the certified ELISAs.

Using Equation (2), the mean bias of measuring serum ferritin with the PC biosensor was 7 ng/mL higher than when measuring it on the ELISA. These results are better visualized in the ferritin difference plot (Figure 2A). When measuring ferritin, the inherent imprecision (σ(δ)) of both diagnostic methods was 24 ng/mL using Equation (1). Inherent imprecision indicates the range in which the mean differences must fall in order to fail to reject the null hypothesis that there are no differences between methods. That is, 68% and 95% of the differences must fall between 0 ± 24 and 0 ± 48 ng/mL, respectively. The actual distribution of mean differences was 43% at 0 ± 1σ(δ) and 68% at 0 ± 2σ(δ). Thus, in the case of ferritin, the PC assay’s inherent analytical imprecision was different than that of the ELISA.

A similar analysis for sTfR using Equation (2) resulted in an average bias of −0.09 µg/mL when comparing the PC biosensor against the ELISA. The inherent imprecision of the PC and ELISA was σ(δ) = 0.23 µg/mL (Figure 2B). Therefore, these data denoted that 68% of the sTfR differences between the PC biosensor and ELISA fell between 0 and 0.23 µg/mL, and 95% of differences fell between 0 and 0.46 µg/mL. The actual distribution was 62% at 0 ± 1σ(δ) and 96% at 0 ± 2σ(δ). Therefore, based on the inherent imprecision test for sTfR, there was no statistical difference between the methods.

### 3.3. Determination of Systematic Error and Random Error

**Figure 3:**
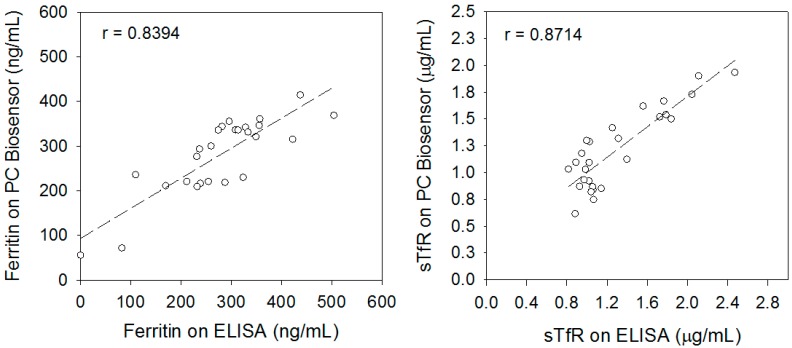
Comparison plots showing the analytical range and Pearson correlation coefficient of ferritin and sTfR measured in sera of hemodialysis patients using the PC biosensor and ELISAs.

The statistical differences in bias and random error were calculated. Although least-squares regression analysis is preferred to compare methods and determine bias over a wide analytical range, paired *t*-test and F-test statistics were used. This is because the linear correlation coefficient (r) obtained from the results of the PC and ELISA was less than 0.99, as shown in the comparison plots (Figure 3). In this case, the estimates of the y-intercept (i.e., bias) and slope (proportional error) are not reliable, and thus it is better to use paired *t*-tests for calculations [37]. It is possible that the limited analytical range of samples used in this study as shown in the comparison plots (Figure 3) might have affected the r values. Based on the paired *t*-test (Equation 4), there was no bias in determining ferritin using the PC biosensor in comparison to ELISA test (*t*-value = 0.87 vs. *t_critical_*-value = 2.06; *p* > 0.05). This was not the case for sTfR (*t*-value = 2.23 vs. *t_critical_*-value = 2.05; *p* < 0.05), in which systematic error was observed. Despite the empirical usefulness of paired *t*-tests to evaluate systematic error, these results can be misleading if not interpreted correctly. Referring back to Equation (4) when rearranged, it is observed that a large SD_diff_ can lead to a small *t*-value (e.g., in the case of ferritin). Likewise, a large bias term or $\sqrt{N}$ term can lead to large *t*-values. Therefore, it is important to consider the clinical significance of these errors when diagnosing patients, not solely whether a systematic error exists. 

There were differences in random error (F-test, Equation (5)) between bioassays when measuring ferritin, but not sTfR. Random error for the PC biosensor was higher than the ELISA test when measuring ferritin (F = 4.38 vs. F-value = 2.06; *p* < 0.05). For sTfR, the random error was similar for both methods (F = 1.62 vs. F-value = 1.93; *p* > 0.05). The high random error values in the ferritin PC assay could be related to differences in ferritin–fAb-ION interactions during magnetic separation compared to those for sTfR–fAb-ION separation. Such differential interactions could have resulted due to the size of ferritin (more than four times the molecular weight of sTfR) as well as the high concentrations of other metabolites in the sera of patients with CKD [31,32,33].

### 3.4. Total Calculated Analytical Error Compared to Total Allowable Analytical Error

Each biomarker has a different quality specification for total allowable error [29]. Total error was calculated using Equation (8) and results were compared to the TEa for each IDA biomarker (Table 2). Based on the National Health and Nutrition Examination Survey (NHANES) 2003–2010, the cutoff for sTfR in healthy populations is 5.3 µg/mL [46]. Although hemodialysis patients with CKD are not considered “healthy,” no quality specification for IDA biomarkers in CKD patients exists. Therefore, the clinical cutoff used by NHANES was applied. For sTfR, TEa (17.6%) was estimated by biological variation studies conducted previously [47]. Because the TEa of sTfR is a percentage, the absolute concentration observed in this study (TEcalc = 0.52 µg/mL) was converted to a percentage of the clinically relevant cutoff (5.3 µg/mL). Thus, the TEcalc of the PC biosensor measuring sTfR was 9.8%, which is lower than the TEa at the clinical cutoff of 5.3 µg/mL. This demonstrated that the performance of the PC compared to ELISA resulted in less error than would be observed in biological variation within and between individuals. However, because the range of sTfR concentrations in the experiments was between 0.6 and2.5 µg/mL in the CKD patients, and did not include 5.3 µg/mL, it is inappropriate to extrapolate the error outside the range that was measured in the experiment. In that case, it is recommended to use the mean of the measured samples instead of the clinical cutoff to determine the TEcalc percentage.

The mean of the measured samples was 1.21 µg/mL, and thus the TEcalc using Equation (8) was 43% (i.e., 0.52/1.21 × 100). Contrary to the TEcalc of 9.8% when using the clinical cutoff of 5.3 µg/mL, the TEcalc of 43% is not less than the established TEa of 17.6% [47]. Although the TEcalc was greater than what is allowed, the clinical decision to diagnose IDA is not affected because all sTfR values are below the cutoff at which deficiency would be suspected (i.e., 5.3 µg/mL).

For ferritin, the TEcalc of the PC biosensor was 94 ng/mL. As a percentage of the mean found in the experimental data, the TEcalc was 33.6%. Previous biological variation studies demonstrate that a TEa of 16.9% is an acceptable amount of error. Therefore, the observed TEcalc was greater than the TEa. As in the case of sTfR, it is important to consider the clinical relevance of this error in the context of how the diagnosis and treatment may change. 

### 3.5. Diagnosis Considerations in Method Validation

This study showed that ferritin was a better indicator than sTfR to identify patients who needed IV iron treatment. Although the two patients with low ferritin (i.e., <100 ng/mL) had higher values of sTfR, no sTfR values exceeded the clinical cutoff (i.e., >5.3 µg/mL) for iron deficient erythropoiesis. In this particular data set, the majority of individuals had ferritin levels between 200 and 500 ng/mL. The National Kidney Foundation stated that ferritin levels >200 ng/mL are ideal to lower the risk of an absolute iron deficiency. To ensure EPO therapy is not limited by iron, the serum ferritin cutoff at which IV iron treatment is recommended for CKD patients is <100 ng/mL. After applying this cutoff to our data, from both diagnostic platforms, it was found that the same hemodialysis patients with CKD should receive IV iron therapy to prevent the consequences of an iron-deficient erythropoiesis that ultimately would lead to IDA. 

sTfR values from all samples measured on either platform were not above or near the cutoff indicative of deficiency. Values were similar to those reported in other studies with CKD patients [15,43,44,45]. Low sTfR levels may be the result of patients who had recently received EPO. This results in a rapid drop in ferritin levels, as its cargo is now used in hemoglobin synthesis before sTfR is upregulated [15]. Nevertheless, more measures of iron status such as TSAT would be needed to reach a reliable diagnosis of iron deficiency [10]. More studies are needed to elucidate the value of circulating sTfR as a valid biomarker of IDA in CKD patients. 

The process of method validation undertaken in this study was derived from the Stockholm Consensus Conference held in 1999, in which global analytical quality specifications were set [49] and had since been updated yearly based on ongoing studies collecting biological variation data of new biomarkers [29]. Quantifying the total analytical error and comparing it to established quality specification is essential when evaluating new diagnostic platforms. The method validation process is long and labor-intensive, yet it is critical considering that clinical diagnoses have treatment consequences for patients. High-end validated clinical diagnostic instruments (e.g., Centaur XP and Abbott Architect i2000) have been rejected for the measurement of certain analytes due to unacceptable analytical errors [50]. Holmes and colleagues (2013) contrasted 25-hydroxy-vitamin D in 160 specimens measured by both the Centaur and Architect analyzers as the test methods against liquid chromatography mass spectroscopy and a radioimmunoassay as reference methods. Due to unacceptable levels of random variability and a high-degree of positive systematic error (potentially due to a suspected interfering antibody), both test methods (i.e., Centaur and Architect) did not meet the TEa when measuring 25-hydroxy-vitamin D [50]. Regardless of portability, POC technologies need to undergo similar scrutiny before being launched as dependable clinical diagnostics [51]. Although a rapid turn-around time is important in POC diagnostics, accuracy and reliability supersede turn-around time due to the potential consequences of misdiagnosis in at-risk populations. As new biosensors continue to penetrate the clinical and outpatient markets, it is critical that a standard method validation process is followed to ensure proper evaluation of analytical performance.

### 3.6. Limitations

Based on the total analytical error observed when measuring IDA biomarkers on the PC biosensor, optimization is required to meet current quality specifications. In this regard, minimal optimization of the PC has been conducted. Steps to reduce the TEcalc of the PC biosensor include adding wash steps during magnetic separation, adjusting capture and detection antibody concentrations, and changing dilution buffers, which may enhance fAb-ION stability. Assay time may be reduced by following fAb-ION–antigen binding using dynamic light scattering instruments and determining the minimum time required for fAb-ION and antigen association. In addition, functionalizing protein A or streptavidin to the IONs and conjugating the detection antibodies with a predictable orientation may improve selective binding and decrease non-specific binding near the fAb-IONs surface. Furthermore, depending on the target population for using the PC biosensor for clinical diagnosis, hemodialysis patients may not have been the best population to perform method validation experiments using fAb-IONs because they are known to have various metabolic byproducts and wastes in their sera. These affected the colloidal stability of fAb-IONs and antigen complexes, resulting in a lesser number of samples used in the final validation analysis. Presence of serum proteins and other biological metabolites have been shown to affect the colloidal stability of IONs [52,53].

## 4. Conclusions

This is the first thorough method validation study quantifying total analytical error using a PC biosensor for nutrient biomarkers and comparing it to established quality specifications. Although previous studies showed excellent performance of the PC biosensor in quantifying sTfR using WHO standard reference materials against certified ELISAs, the interference molecules in CKD samples resulted in significant analytical error. Nevertheless, similar to the certified ELISA, the diagnosis in hemodialysis patients with end-stage renal failure with the PC biosensor indicated that more than 90% of the patients in the sample had sufficient iron stores. Future studies will aim at optimizing the protocol of the PC biosensor so as to reduce total analytical error and expand the number of biomarkers that can be measured from the same specimen.

## Figures and Tables

**Table 1:** Performance, means, SD, coefficient of variations, and range of measurements for all biosensing platforms and analytes.

Sensing Platform		Mean	SD	CV (%)	Range	LOD ^c^	Dynamic Range
sTfR ^a^	PC Biosensor	1.21	0.16	12.8	0.62–1.93	21	20–500
	R&D ELISA	1.30	0.15	11.2	0.82–2.47	42.5	255–6800
Ferritin ^b^	PC Biosensor	279.5	23.7	8.5	56–414	26	26–2000
	ALPCO ELISA	276.8	11.3	4.1	0–504	5	15–1000

^a^ sTfR measurements are in µg/mL for both platforms; ^b^ Ferritin measurements are in ng/mL for both platforms. For ELISA, according to vendor specifications; ^c^ LOD and dynamic range in ng/mL. For PC biosensor, LOD was calculated as in [42]. CV: coefficient of variation; LOD: limit of detection; SD: standard deviation.

**Table 2:** Total calculated analytical error (TEcalc) for iron deficiency anemia (IDA) biomarkers measured on the PC biosensor against established quality specifications for total allowed error (TEa).

Biomarker	n ^1^	TEcalc ^2^	TEcalc ^3^ (%)	TEa (%)	TEcalc ^4^ (%)
sTfR (µg/mL)	27	0.52	43.0	17.6 ^5^	9.8
Ferritin (ng/mL)	23	94	33.6	16.9 ^6^	n/a

^1^ Number of patients included in analysis; ^2^ TEcalc as absolute concentration; ^3^ TEcalc as a percentage; ^4^ TEcalc for clinical cutoff at 5.3 µg/mL; ^5^ TEa cited by Bailey et al., 2014; ^6^ TEa cited on Westgard’s online database [48].
